# Evaluation of the application of sequence data to the identification of outbreaks of disease using anomaly detection methods

**DOI:** 10.1186/s13567-023-01197-3

**Published:** 2023-09-08

**Authors:** José Manuel Díaz-Cao, Xin Liu, Jeonghoon Kim, Maria Jose Clavijo, Beatriz Martínez-López

**Affiliations:** 1 Center for Animal Disease Modeling and Surveillance (CADMS), Department of Medicine & Epidemiology, School of Veterinary Medicine, University of California, Davis, USA; 2 Departamento de Patoloxía Animal, Facultade de Veterinaria de Lugo, Universidade de Santiago de Compostela, Lugo, Spain; 3 Department of Computer Science, University of California, Davis, USA; 4 Department of Veterinary Diagnostic and Production Animal Medicine, College of Veterinary Medicine, Iowa State University, Ames, USA

**Keywords:** Outbreak detection, machine learning, regression, control chart, surveillance, epidemics, production system, animal health

## Abstract

Anomaly detection methods have a great potential to assist the detection of diseases in animal production systems. We used sequence data of Porcine Reproductive and Respiratory Syndrome (PRRS) to define the emergence of new strains at the farm level. We evaluated the performance of 24 anomaly detection methods based on machine learning, regression, time series techniques and control charts to identify outbreaks in time series of new strains and compared the best methods using different time series: PCR positives, PCR requests and laboratory requests. We introduced synthetic outbreaks of different size and calculated the probability of detection of outbreaks (POD), sensitivity (Se), probability of detection of outbreaks in the first week of appearance (POD1w) and background alarm rate (BAR). The use of time series of new strains from sequence data outperformed the other types of data but POD, Se, POD1w were only high when outbreaks were large. The methods based on Long Short-Term Memory (LSTM) and Bayesian approaches presented the best performance. Using anomaly detection methods with sequence data may help to identify the emergency of cases in multiple farms, but more work is required to improve the detection with time series of high variability. Our results suggest a promising application of sequence data for early detection of diseases at a production system level. This may provide a simple way to extract additional value from routine laboratory analysis. Next steps should include validation of this approach in different settings and with different diseases.

**Supplementary Information:**

The online version contains supplementary material available at 10.1186/s13567-023-01197-3.

## Introduction

Animal diseases are responsible for annual production losses estimated at more than 20% [1], as well as millions of expenditures in disease control [2]. Thus, to minimize animal and economic losses, proactive approaches focused on prevention, monitoring and early intervention are vital and strongly encouraged to maintain the sustainability of the livestock sector [3]. Several efforts have attempted to implement systems for the timely detection of outbreaks in both human and animal health [4–7]. This may be accomplished by monitoring real-time or near-real-time, time series of parameters that are indicative of the presence of disease (e.g., mortality, occurrence of clinical signs or laboratory results) [8]. In this context, a disease outbreak can be understood as an anomaly in the normal disease background (either absence or endemic presence of a disease) of the population. For example, syndromic surveillance (SyS) uses non-specific, prediagnostic health parameters such as clinical signs or other indirect indicators of disease (mortality, decrease in animal production, etc.) to detect disease events [4, 8–10] This approach has proven to be cost-effective and has been increasingly applied in animal health in the last decade [4, 5, 11].

Alternatively, laboratory results can be used to monitor the temporal patterns of specific pathogens [12]. The use of these data for monitoring is not without limitations in terms of data availability, population coverage, and timeliness [5], but can be helpful when dealing with groups of farms affected by specific diseases. Some livestock sectors, such as swine production, are highly integrated systems. In these, individual farms have an agreement with a corporation in which individual farms provide facilities and personnel, but the animals are owned by the corporation, which in turn is responsible for providing food, meds, veterinary services, and technical advice. Therefore, farms under the same integrating corporation or production system are usually highly connected (e.g., through feed trucks or animal movements), which facilitates the rapid spread of diseases. Therefore, if we can detect outbreaks in the system early, we can prevent the spread of diseases into naïve farms in the same system.

To monitor anomalies and detect outbreaks different laboratory data can be used, e.g., positive results, laboratory requests, etc. However, these data may have limitations for detecting outbreaks in endemic scenarios, since case counts may be the result of the endemic presence of a pathogen and not a disease emergency. In this situation, sequencing is useful to differentiate new strains from those already circulating and, therefore, to distinguish between endemic or epidemic scenarios. The combination of time series of sequencing data with anomaly detection methods could help detect the emergence of new strains in a production system and prevent their further spread through farms in the system. However, these approaches have not been extensively explored as there are two traditional main limitations: the limited use of sequencing in livestock productions, and the lack of development of anomaly detection methods for this type of data. Regarding the first one, the use of sequencing has increased significantly in recent years, especially for certain diseases of economic importance such as Porcine Reproductive and Respiratory Disease (PRRS) [13]. This is a viral disease associated to large losses (more than $600 million a year in the USA alone [14]), with no pathognomonic signs, and endemic in most major pork-producing countries. Porcine Reproductive and Respiratory Disease virus (PRRSV) has a high viral mutation rate, it can reassort with vaccine strains, and commercial vaccines may not fully protect against new strains [15, 16]. This can lead to a rapid emergence of new strains from inside or outside the farm. Therefore, routine sequencing of PCR products is increasingly requested to assess whether a farm is experiencing the presence of a novel wild-type strain, a vaccine strain, or an endemic strain. Indirectly, this provides a source of sequenced data that could be used for anomaly detection [14].

In order to apply anomaly detection methods to sequenced data, it is also necessary to evaluate their performance. We can find a vast number of anomaly detection methods applied to different surveillance systems [17, 18]. In general, the most common anomaly detection methods can be mainly grouped into (i) regression models, using modifications of the Serfling’s approach [19]; (ii) time series analysis: Holt-Winters exponential smoothing (HW) or Autoregressive Integrated Moving Average models (ARIMA); and (iii) detection algorithms inspired by statistical process control methods or control charts (SCC): Shewhart charts, Early Aberration Reporting System (EARS), Cumulative Sums (CUSUM) or Exponential Weighted Moving Average (EWMA). Various studies have attempted to assess the selection of a method, but the performance of each of them can be very variable depending on the situation [8, 20]. Several factors related to the characteristics of the time series (frequency, variance, secular trends, seasonalities, length of baseline data, etc.), distribution of data or characteristics of the epidemic (e.g., amplitude, duration, and diversity of the outbreaks) may compromise the accuracy of a method. The choice of the specific algorithm must also consider the specific type of data and surveillance objectives [4, 8, 17, 20]. Moreover, statistical methods may require human intervention to correctly parameterize each time series, which may not be an easy task [21]. From a practical point of view, an adaptable algorithm that does not need constant human supervision would be more desirable as today’s surveillance systems are usually automated [5, 22]. In this regard, machine learning approaches may offer more adaptive and robust anomaly detection systems by learning from the available data. However, even though machine learning has been successfully applied to the detection of anomalies in diverse types of times series, including space shuttle or power demand [23]; the application of these approaches in animal health surveillance is currently limited.

In this study we aim to evaluate the use of sequence data for early detection of disease using anomaly detection methods. To our knowledge, this has not been attempted before with this type of data. Thus, we first evaluated the performance of 24 anomaly detection methods to detect outbreaks in time series of new strains defined from sequence data. Then, in order to compare the gain of using sequence data, we selected the best methods in this evaluation to compare their performance using other types of data: positive PCR counts, PCR requests counts and laboratory requests counts. For illustration purposes, we used PRRSV sequences and laboratory data from a large swine production system in the US. With this, we expect to provide a picture of the different capabilities of anomaly detection in sequence data to the early detection of disease.

## Materials and methods

### Data collection

We collected 63 671 individual laboratory records consisting of sample submissions for different types of analysis (ELISA, PCR, sequencing, etc.) for PRRSV. Submissions were made between 2016 and 2020 by a vertically integrated swine production system in which PRRS is endemic. Data were recorded on the Disease BioPortal platform [24], which presents different functionalities for the management and analysis of animal health data. The production system is made up of sites dedicated to different operations: breeding/gestation, farrowing, nursery, wean-to finish, farrow-to-finish, etc.; located in the United States. Farm names, locations, and characteristics are not provided for confidentiality reasons.

### Data management

We aggregated the individual laboratory records by week and farm to create a time series of counts of the number of farms that requested at least one diagnosis of PRRS of any type, each week (*n* = 10 103 weekly laboratory requests). The result was a time series composed of 209 weeks from 2016 to 2020. We chose a weekly aggregation because the volume of diagnostic data generated in a production system is not usually high on a daily basis. Furthermore, weekly aggregation has demonstrated good performance in previous studies [25]. Next, we filtered the time series by the farms that requested PCR analysis (*n* = 5798 weekly PCR requests farms). We then filtered this time series to count those farms that tested positive to PCR in at least one individual sample (*n* = 1995 weekly PCR positives farms).

Finally, among the counts of positives, 928 submissions had sequence information and were used to assess the novelty of a sequence in each farm and construct a time series of new strains. We considered that a sequence represents a new strain in a farm when the following criteria were met: (i) the sequence was different from a previously sequenced strain from the same farm; and (ii) the sequence was different from vaccine strains. The differences between strains were analyzed by phylogenetic analysis using the neighbor joining method. We included all the obtained field strains in it as well as reference sequences for the following vaccines: Ingelvac PRRS ATP (Boehringer Ingelheim, Germany), Ingelvac PRRS MLV (Boehringer Ingelheim, Germany), Fostera PRRS (Zoetis, USA) and Prime Pac PRRS (MSD, USA). The analysis was performed using the correspondent tool available in the Disease BioPortal. To consider a sequence different, it must have presented a distance greater than 0.03 from previous strains or from vaccine strains. We established this value as a threshold, as it is commonly used in previous studies to identify new PRRSV field isolates [26]. In each week we counted a farm as positive to a new strain when the farm presented at least one new wild-type sequence in that week. This resulted in the detection of new strains in 751 PCR-positive farm weekly submissions out of 928.

**Figure 1:**
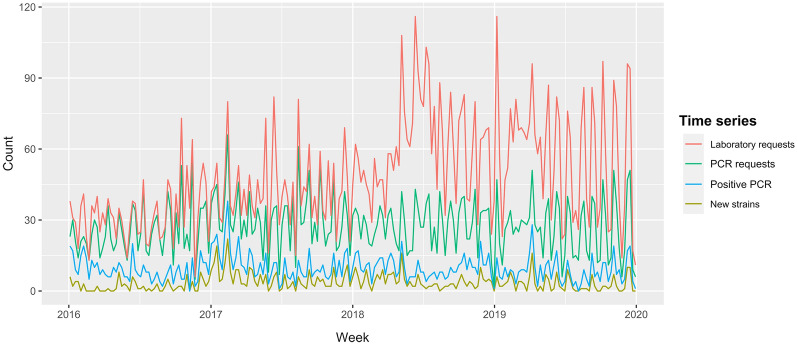
**Time series of the weekly counts (2016–2020) of new strains of PRRSV, PCR positives, PCR requests and laboratory requests in the production system.**

In consequence, we obtained four different time series of counts: laboratory requests (i.e., counts of any type of diagnostic requested to detect PRRSV or antibodies), PCR requests, PCR positives and new strains (Figure 1).

### Anomaly detection algorithms and outbreak simulation

We conducted a comprehensive review of anomaly detection methods used for human and animal health surveillance in the literature and ultimately included 24 methods in this evaluation. The characteristics, family type, thresholds, and tuning parameters of each method are shown in Tables 1, 2 and 3 along with the abbreviations used in this paper and the software and functions used to run each model. The types of the methods we used were: regression (Farrington’s algorithm (*far*), improved Farrington’s algorithm (*farflex*), negative binomial regression (*breg*)), machine learning (Long Short-Term Memory (LSTM) (*lstm*)), Bayesian approaches (*bay1–3*), time series methodologies (ARIMA (*ari_g/ng*) and HW (*hw_g/ng*) and SCC algorithms (RKI algorithm (*rki1–*3), EARS algorithm (*ears1–3*), CUSUM and Shewhart charts with different pre-processing techniques (*lstm_c/s*, *breg_c/s*, *far_c/s*) and EWMA (*ewma*)). We present more information about the general principles of each type of method evaluated in the Additional file 1.

**Table 1:** **Anomaly detection methods evaluated based on machine learning, regression techniques and Bayesian analysis, thresholds to detect outbreaks by each method and tuning parameter used.**

Name	Method abbreviation	Threshold	Software/references	Parameters
Machine learning				
A deep learning algorithm based on Long Short-Term Memory (LSTM)	*lstm*	Two times the standard deviation of the residuals	Implemented with Python using the packages *scikit-learn* [55] and *tensorflow* [56]	Prediction window = 10 Layer LSTM = 3 (None, 50, 10) (None, 50, 10) (None, 10) Layer dense = (None, 1) Optimizer = adam Epochs = 100
Regression techniques				
Original Farrington’s algorithm	*Far*	2/3 power transformation of the Poisson distribution in both methods	Implemented in R as described by the author using the package *surveillance* using functions Farrington() and farringtonFlexible(), respectively [21, 27]	Training window (years) = 2 Window size = 4 *α* = 0.025
Improved Farrington’s algorithm	*farflex*			Training window (years) = 2 Window size = 4 Threshold for reweighting = 2.58 *α* = 0.025
Negative binomial regression including trend and seasonality	*breg*	two times the standard deviation of the residuals	Implemented in R using the package *glmmTMB* [57]	$\mathrm{log}\left(y\right)={B}_{0}+t+sin\left(\frac{2\pi t}{52}\right)+cos \left(\frac{2\pi t}{52}\right)$ *t* = trend (from 1 to 157)
Bayesian analysis				
Bayes algorithms	*bay1*	(1 − *α*) * 100% quantile (95%) of the posterior distribution	Implemented in R using the package *surveillance* using the functions algo.bayes1, algo.bayes2 and algo.bayes3, respectively [58, 59]	Ref. values = 6 previous weeks; *α* = 0.05
	*bay2*			Ref. values = 6 previous weeks and 13 weeks previous year; *α* = 0.05
	*bay3*			Ref. values = 9 weeks from 1 year ago and 9 weeks from 2 years ago; *α* = 0.05

**Table 2:** **Anomaly detection methods evaluated based on time series techniques, criteria to detect outbreaks and tuning parameters.**

Name	Method abbreviation	Threshold	Software/references	Parameters
Holt-Winters exponential smoothing with (*hw_g*) and without guard-band (*hw_ng*)	*hw_g* *hw_ng*	Two times the standard deviation of the residuals	Implemented in R using the package *forecast* [60]	(additive, none, none)
ARIMA with (*ari_g*) and withoutt guard-band (*ari_ng*)	*ari_g* *ari_ng*	Two times the standard deviation of the residuals	Implemented in R using the package *tscount* [61]*.* We evaluated the need of a previous differencing step. To select the adequate parameters for the ARIMA models, we ran models with all the possible combinations for the ARMA parameters from zero to four, and selected the best model based on the Aikake’s information criterion	ar = 4; d = 0; ma = 1

**Table 3:** **Anomaly detection methods evaluated based on control chart algorithms, criteria to detect outbreaks and tuning parameters.**

Name	Method abbreviation	Thresholds	Source/Implementation	Parameters
RKI algorithms	*rki1*	The observed value exceeds a confidence interval (*α* level of confidence) of the mean of the reference values (weeks)	Implemented in R using the package *surveillance* using the functions algo.rki1, algo.rki2 and algo.rki3[58]	Ref. values = 6 previous weeks; *α* = 0.05
	*rki2*			Ref. values = 6 previous weeks and 13 weeks previous year; *α* = 0.05
	*rki3*			Ref. values = 9 weeks from 1 ago and 9 weeks from 2 years ago; *α* = 0.05
EARS algorithm C1	*ears1*	The observed value exceeds 1 − *α* quantile of the standard normal distribution	Implemented in R using the package *surveillance* using the function earsC(). The arguments C1, C2, and C3 select the different methods [58]	Baseline = 7 weeks; *α* = 0.05
EARS algorithm C2	*ears2*			Baseline = 7 weeks; *α* = 0.05
EARS algorithm C3	*ears3*			Baseline = 7 weeks; *α* = 0.05
CUSUM	*lstm_c* *far_c* *breg_c*	Two times the standard deviation of the reference values	Pre-processing method were performed as described above (*lstm, far* or *breg*). The control charts were implemented in R using the package *qcc* [62]	Decision interval = 2 × standard deviation
Shewhart	*lstm_s* *far_s* *breg_s*			conf = 2
EWMA	*ewma*	Two times the standard deviation of the reference values	Implemented in R using the package *otsad* [63, 64]	Smoothing constant = 0.01, control limit multiplier = 2

The performance of the algorithms was evaluated by generating and injecting synthetic outbreaks into the time series as described previously [27]. A synthetic outbreak was composed of a set of consecutive weeks in which synthetic cases were added to the original time series of counts. The *outbreak size* was defined as the sum of the cases in the synthetic outbreak, so it is made up of the original counts plus the added synthetic cases.

**Figure 2:**
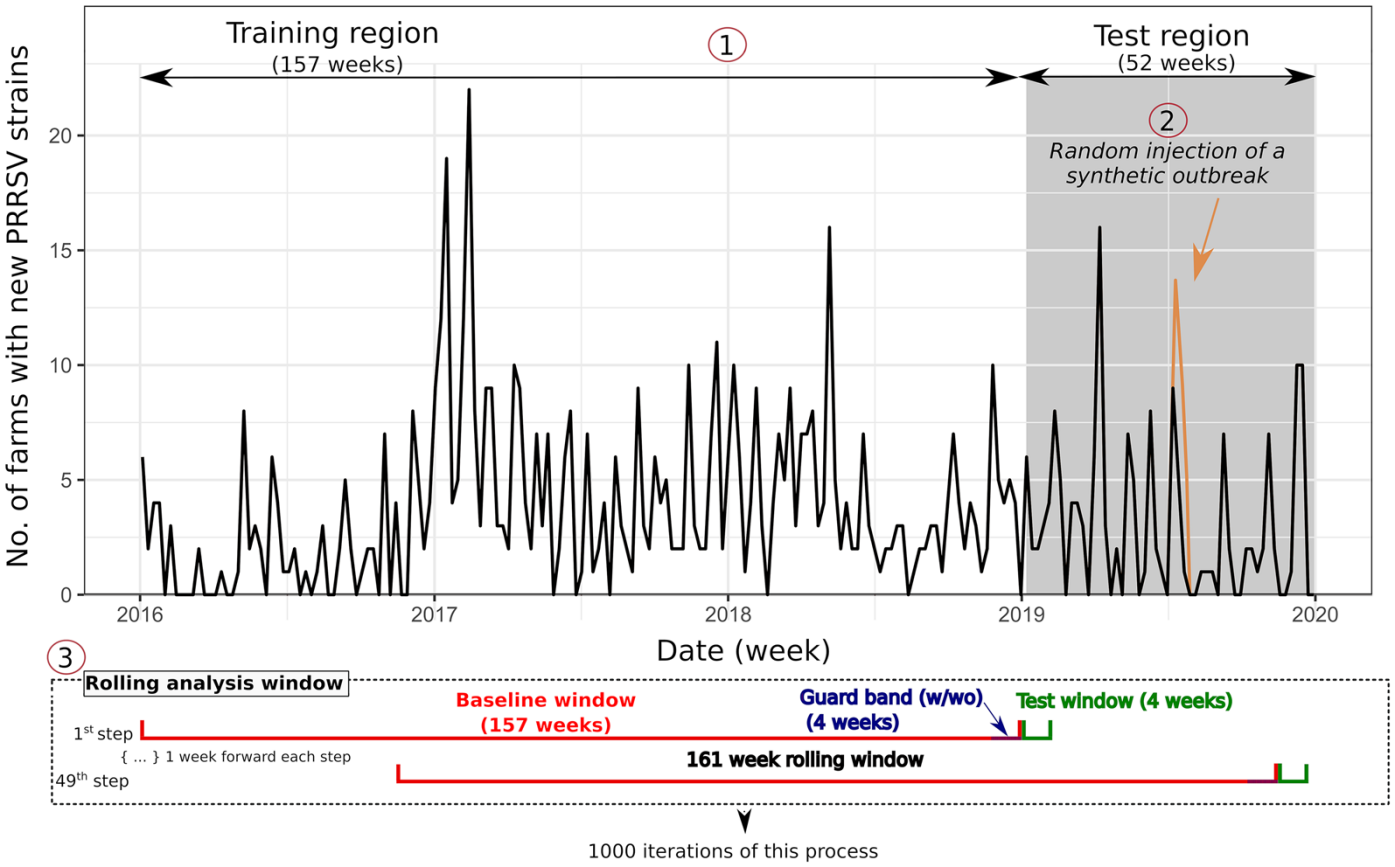
**Scheme of the evaluation process for each anomaly detection method using the time series of the weekly counts of new strains of PRRSV in the production system as example.** (1) The complete time series consisted of 209 weeks divided into a training region (157 weeks) and a test region (52 weeks). (2) At each iteration, a synthetic outbreak was randomly injected into the test region. (3) For each iteration, the analysis was performed in rolling windows of 161 weeks divided into 157 weeks for the baseline window and 4 weeks for the test window. The analysis was repeated pushing the 161-week window forward 1 week until the last week of the test window reached week 209 (49 steps). The process of injection of synthetic outbreaks and subsequent rolling analysis was repeated in 1000 iterations for each algorithm. The evaluation was made with and without a 4-week guard band between the baseline window and the test window.

A synthetic outbreak was created using three parameters: the number of synthetic cases, the number of consecutive weeks that will constitute the outbreak and the distribution of the synthetic cases in those weeks. The synthetic outbreaks generation and injection process consisted of: (1) We randomly selected 1 week from the last 52 weeks of the time series (test region) (Figure 2). That week was considered the beginning of the outbreak. (2) We generated the total number of synthetic cases that composed the outbreak. This was done using a Poisson random variable of mean equal to *k* times the standard deviation of the baseline window, the first 157 weeks of the analysis window of the time series (Figure 2). (3) The synthetic cases were distributed in time following a lognormal distribution (mean = 0; standard deviation = 0.5) [27]. (4) Finally, the synthetic cases were summed to the original time series starting in the week selected at the step 1 and distributed according to the distribution obtained in the step 3.

This outbreak generation process creates randomness in the week of the beginning of the outbreak, in the duration of synthetic outbreaks, and in the number of synthetic cases that compose the synthetic outbreak. This is intended to represent the variability of an outbreak in field conditions. The size and duration of outbreaks are modulated by the parameter *k*, so that increases in *k* increase the number of cases and length of the outbreak. Thus, to explore the ability of each algorithm to detect outbreaks of different magnitude, we used different values of *k*: 4, 8, 12, 16 and 20. Our intention was simply observing the trends of the behavior of the algorithms, so the selection of these *k* values was arbitrary. The variation in number of synthetic cases added and duration of outbreak by *k* value are showed in Additional files 2 and 3.

### Analytical approach and anomaly detection algorithm evaluation

We analyzed the 209-week time series in a rolling analysis window of 161 weeks composed of two parts: a 157-week baseline window, used as reference for the algorithms; and a 4-week test window, in which the algorithms tested for any anomaly. This interval of 4 weeks was chosen since it is considered that PRRS virus may spread to all production stages and produce clinical outbreaks in about 2–3 weeks [28], so we expected this period to be enough to observe an outbreak of PRRS. We repeated the analysis moving the analysis window 1 week forward each time until completing the whole time series (Figure 2) (49 steps). The analysis was repeated in 1000 iterations for each anomaly detection method and *k* value. Each iteration consisted of a generation and injection of the synthetic cases in the testing region as described before, followed by the rolling analysis of the 49 analysis windows to evaluate if the algorithm was able to detect the injected outbreak. In addition, we considered the inclusion of a guard band of 4 weeks between the baseline window and the test window (Figure 2) (45 steps in this case). The guard band is not evaluated, but it creates a gap between the training and the test windows to avoid the contamination of the baseline window with a potential early phase of an outbreak [29]. Each anomaly detection method was evaluated with and without this guard band. However, for simplicity, we only presented results of methods with a guard band in the “Results” section when the addition of the guard period caused a substantial change in the results (more than 5% of variation in any performance measure).

We used the term *signal* to denote the individual weeks with synthetic cases that composed the synthetic outbreak, and the term *alarm* to refer a detected anomaly i.e., when the number of cases crossed the threshold determined by an anomaly detection method. The outbreak was considered detected if an alarm was generated at least once between the start and the end of an outbreak [27]. The performance of the algorithms was evaluated using four measures:

- Sensitivity (Se), which refers to the percentage of the sum of all the signals detected by the method over the sum of total signals injected in all the iterations, i.e., detections in each week with true outbreaks. This measures the ability of the algorithm to detect all the anomalies. It is an observation-based sensitivity.
- Probability of detection (POD), which is the percentage of outbreaks detected of all the iterations. This measures the ability of the algorithm to detect at least one anomaly for each outbreak and thus identify the outbreak. It is an event-based sensitivity.
- Probability of detection in the first week (POD1w), which is the proportion of the detected outbreaks in which the alarm occurred in the first week, i.e., how many outbreaks are detected in the first signal. This measures the timeliness of the algorithm.
- The background alarm rate (BAR), which is the proportion of alarms triggered in weeks with no synthetic outbreaks. We used this parameter instead of specificity because there was no information available about the real occurrence of outbreaks in the production system [30]. Thus, the calculation of specificity is not possible since we cannot rule out that natural outbreaks may be present in the observed data.

In order to have a metric that provided a general measure of the overall performance of the methods, we defined an overall score:

$$\mathrm{Overall score}=\mathrm{POD}+\mathrm{Se}+\mathrm{POD}1\mathrm{w}$$

### Comparison of anomaly detection performance with time series made with other data

After evaluating the time series of new strains with the 24 anomaly detection methods, we selected those methods that performed the best to use them with the other three time series created in our data management flow (PCR positives, PCR requests and laboratory requests (Figure 1)). This allowed us to compare the performance of the algorithms in different scenarios and explore the value of using sequenced data to detect outbreaks.

The methods we selected were the best method for each of the parameters POD, Se and POD1w; and the three methods that presented the best overall score (Figure 4), ensuring that they were methods of different types (machine learning, regression, etc.). We repeated the same analytical methodology described in the previous section with each time series using the selected anomaly detection methods. Each synthetic outbreak was created using the standard deviation of the correspondent time series and we calculated the same performance measures.

### Software and code

The different anomaly detection methods were run with packages freely available for the statistical software R v. 4.1.1, except LSTM, which was run in Python v. 3.8.8. The names of the packages used for each method are detailed in Tables 1, 2 and 3. The Additional file 4 shows the functions we implemented for the evaluation in rolling windows and can be used with a sample of the data (Additional file 5) and can be used with other data sets. Also, the specific anomaly detection algorithms used in this paper are also provided in Additional file 6 to be run with the Additional file 5 or with other data following the instructions presented along the document.

## Results

### Evaluation of anomaly detection methods using time series of new strains from sequence data

The weekly behavior of the time series of new strains is showed in Figure 2. An irregular seasonal pattern was observed with an increase in the number of counts during the autumn–winter. Moreover, an important change in the trend at the beginning of 2017 was observed along with a high variability between weeks (SD = 3.6) (Figure 2). The number of farms with new strains in each week ranged from zero to 22 throughout the series, with a mean of 3.6 cases and a median of 2.0 per week. Regarding the simulated outbreaks, after 1000 iterations the mean durations were 2.0, 2.5, 3.0, 2.8 and 2.9 weeks, respectively for *k* = {4, 8, 12, 16, 20}. The peak occurred on average at 1.4, 2.0, 2.3, 2.5 and 2.6 weeks, respectively, after the beginning of the outbreak and the maximum numbers of cases injected in the total duration of the synthetic outbreak were 7.6, 10.3, 11.6, 13.2 and 14.2 on average.

**Figure 3:**
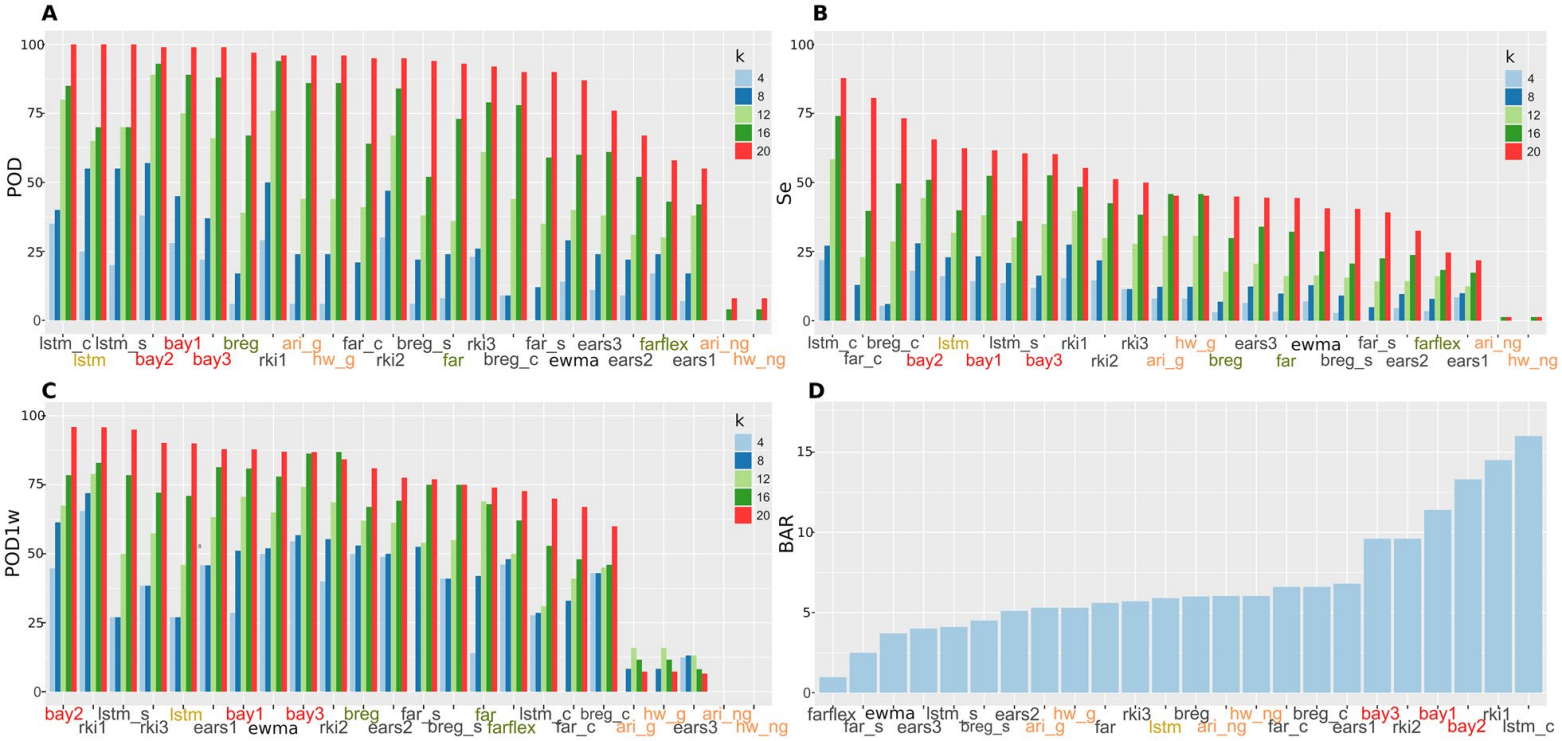
**Results of the performances measures of the 24 anomaly detection methods evaluated for A: probability of detection (POD); B: sensitivity (Se); C: probability of detection in the first week (POD1w) and D: background alarm rate (BAR), for each level of outbreak size (*k*).** Methods are ordered from higher to lower at *k* = 20. For BAR values of different *k* are averaged since they are approximately constant for each *k* value. The color of labels indicates the general type of method: yellow: machine learning; dark green: regression; orange: time series methodology; red: Bayesian; black: control chart.

The values of each performance measure for each anomaly detection method are detailed in Figure 3. The standard error was lower than 2% for each parameter. For most of the methods, the values began to exceed 50% of POD, Se and POD1w when *k* > 12. Regarding POD, the performance was generally poor when *k* was low (*k* < 12), but it was good in large outbreak sizes. For example, when *k* = 12, 9/24 (37.5%) of the methods presented a value over 60% and this performance notably improved as *k* increased: e.g., 13/24 (54.2%) when *k* = 16 and 17/24 (70.8%) when *k* = 20 (Figure 3); and more than 50% of the methods presented a POD higher than 70.0% when *k* = 16 (Table 4). The best methods to detect outbreaks were based on LSTM and Bayesian approaches. CUSUM also performed well, but only when LSTM was used in pre-processing. Sensitivity was generally low though it improved as *k* increased (Table 4). The methods based on CUSUM, (regardless the pre-processing method), LSTM and Bayesian approaches presented the best values. For most of the methods analyzed, POD1w was already higher than 50% for values at *k* = 8 (12/24; 50% of the methods), indicating a moderate overall ability of these methods to detect outbreaks early. Finally, BAR values of different *k* are averaged since they are approximately constant for each *k* value. BAR values were lower than 16% for all methods with a median of 6.0, and lower than 10% for 20/24 methods (83.3%) (Figure 2). The higher numbers of false alarms were found in some of the methods that had a high detection capacity in other parameters (e.g*.*, *lstm_c*, *rki1*, *rki2*, *bay1* and *bay2*).

**Table 4:** **Minimum–maximum and median of the performance measures (expressed in %) of the 24 anomaly detection methods for the probability of detection (POD), sensitivity (Se), probability of detection in the first week (POD1w) and background alarm rate (BAR) for each value of the parameter** ***k*** **in the time series of new strains.**

Performance measure	Minimum–maximum (median)				
	*k* = 4	*k* = 8	*k* = 12	*k* = 16	*k* = 20
POD	0–38.0 (10.0)	0–57.0 (24.0)	0–89.0 (42.5)	4.0–94.0 (70.0)	8.0–100 (94.5)
Se	0–21.0 (7.2)	0–26.8 (11.8)	0–55.9 (24.3)	1.3–70.9 (35.6)	1.3–84.0 (43.3)
POD1w	0–21.5 (15.9)	0–72.0 (51.9)	0–78.9 (54.5)	0–86.9 (70.1)	0–95.9 (77.3)
BAR	1–16 (6)				

**Figure 4:**
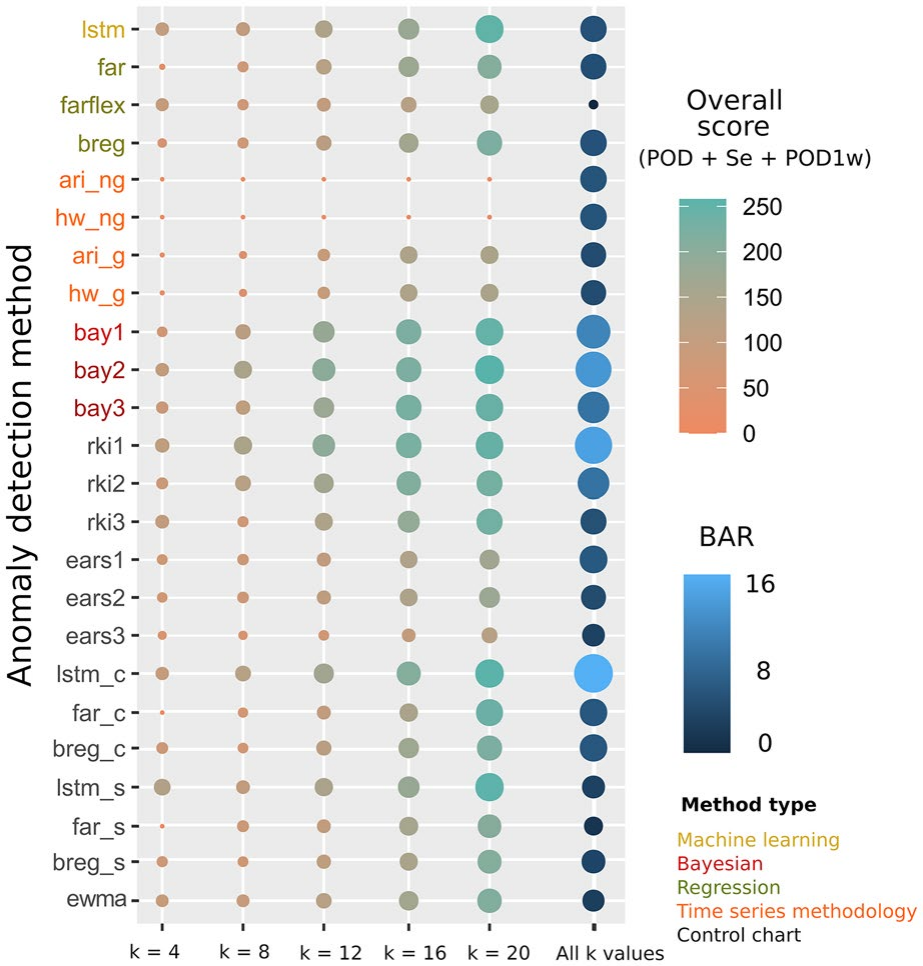
**Overall performance of the 24 anomaly detection methods: sum of the probability of detection (POD), sensitivity (Se) and probability of detection in the first week (POD1w) for each level of outbreak size (*k*) and background alarm rate (BAR) in time series of new strains.** For BAR values of different *k* are averaged since they are approximately constant for each *k* value. Color and size are proportional to values. Labels of each method are presented in different color depending on the type of method: yellow: machine learning; dark green: regression; orange: time series methodology; red: Bayesian; black: control chart.

When considering POD, Se and POD1w together, the *bay* (1–3)*, rki* (1–3) and LSTM-based methods generally presented the best overall scores (Figure 4), followed by *breg*, which presented an intermediate performance in all the parameters (Figure 3). In contrast, methods based on times series techniques and *ears3* presented a substantial worse performance.

Despite conducting all the analysis with and without guard band, the addition of a 4-week guard band did not substantially change the performance of most of the methods under evaluation. For simplicity, only ARIMA and HW are showed in Figure 3, because they were the only ones that did improve their performance when a guard band was added, though they were not among the best methods for any parameter.

### Comparison with other anomaly detection on different types of data

Time series of PCR positives and requests presented a rather similar profile with time series of new strains, but laboratory requests diverged, especially after 2018 (Figure 1). Standard deviations were progressively higher compared to the time series of new strains (3.6 vs 5.5, 11.5 and 22.6, for PCR positives, PCR requests and laboratory requests, respectively) and so the median of counts in each time series (2 vs 8, 27, and 43).

**Figure 5:**
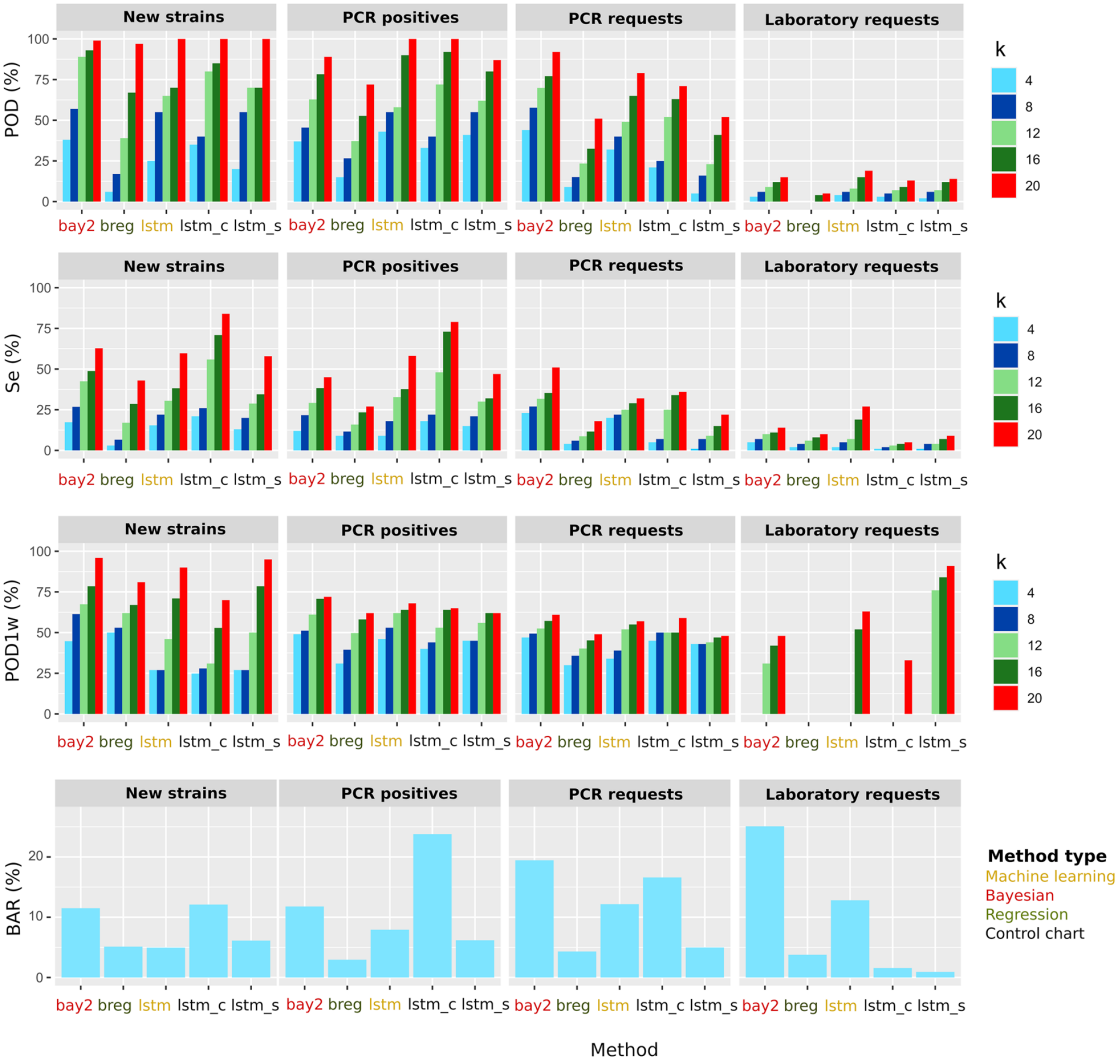
**Comparison of the performance measures: probability of detection (POD), sensitivity (Se), probability of detection in the first week (POD1w) and background alarm rate (BAR); for each level of outbreak size (k) of the selected anomaly detection methods in time series of new strains, PCR positives, PCR requests and laboratory requests.** The color of labels indicates the general type of method: dark green: regression; red: Bayesian; yellow: machine learning; black: control chart. For BAR values of different k are averaged since they are approximately constant for each k value.

Based on the results with time series of new strains, we selected *lstm*, *lstm/c*, *lstm/s*, *bay2* and *breg* and tested them with the other time series. The results of the comparison are presented in Figure 5. In general, detection using sequence data to define new strains performed better than using the other types of data. Differences between new strains and PCR positives were not high, but performance was generally worse when using PCR or laboratory requests (Figure 5). For example, at *k* = 12, the average POD, Se and POD1w were 68.6, 34.9 and 51.3 using sequences; 58.4, 32.6 and 56.4 using PCR positives; 43.5, 19.9 and 47.7 using PCR requests; and 6.2, 6 and 21.4 using laboratory requests, with average BAR values of 9.0, 11.1, 11.5 and 9.4, respectively. Regarding the methods, *breg* and *lstm* showed a balanced performance, although the latter presented too high BAR values when working with PCR and laboratory requests (Figure 5). The algorithm *bay2* systematically presented the higher ability to detect outbreaks and signals, but also showed very elevated BAR values (more than 10% with all the times series).

## Discussion

We present here a systematic evaluation of 24 anomaly detection methods using sequence data and compare their performance regarding the use of different types of data. This is, to the best of our knowledge, the most comprehensive comparison of anomaly detection methods applied to sequence data in livestock to date. The study was designed based on weekly surveillance since livestock production systems usually do not collect diagnostic data daily. Aggregation in weeks also has additional advantages since typical sources of biases of time series, such as the “day of the week” or the “holiday” effect [30] do not need to be taken into account [25, 31]. As a drawback, responses can take up to a week. However, since not all risk events occur on a daily basis (e.g., food deliveries or animal shipments), weekly data still provides adequate time for necessary interventions at the production system level. The evaluation of anomaly detection algorithms in health sciences has been performed on real datasets [32], simulated datasets [33–35] or, as in our study, on real time series for which simulated outbreaks were added [36–38]. Simulated datasets offer more control of how different time series attributes might affect the detection ability of algorithms [39], but they also make assumptions that could deviate from the reality and affect the validity of the inferences. Real background data allow assessing the performance of the algorithms since they include the relevant features that may be present in real datasets (irregular shapes, trend changes, etc.) [12, 36, 38].

The ability to detect outbreaks and signals of most of the methods was good when the *k* parameter increased, as previously observed [36]. However, while POD was high when *k* > 12, the detection of outbreaks was less than 50% at lower outbreak sizes. It must be noted that *k* > 12 implied the addition of more than seven cases in the entire outbreak period (Additional file 2), which may represent the beginning of a significant problem in the system. The worse performance at low *k* values could be explained by the wide variation observed in the baseline data. In general, outbreaks are easier to detect when the incidence and variation of baseline cases are low relative to the outbreak cases [40]. Regarding Se, the values were generally low, suggesting that most alarms were triggered when cases were high enough to be differentiated from the background noise. However, even though several signals went undetected, this circumstance did not result in major problems of timely detection as POD1w was generally high. Thus, large outbreaks could be detected early by various methods with reasonable efficiency. Regarding BAR, previous studies recommend that false alarm rates are fixed at 3% [41] but most of the evaluated methods gave us values just slightly higher, around 5% (Figure 3). Therefore, the presence of false alarms was not considered a big issue. However, some of the methods with higher capability of detection (*bay1–3* or *lstm_c*) also presented too high BAR values. It also must be noticed that, since we did not have information about the presence of outbreaks in the observed data, some of the alarms triggered in weeks in which no synthetic cases were added could be real outbreaks, which could imply a certain overestimation of BAR. We preferred this approach since identifying real outbreaks is challenging even when information is available, as the proper calculation of specificity requires determining the precise onset dates [42].

Most of the approaches typically used in SyS have focused on regression or SCC [5, 42], but we did not find them to have great overall performance in our study, even when different time series were analyzed (Figure 5). In the case of regression, the difficulty of meeting the necessary assumptions about data distribution and independence of observations could contribute to this low performance. Also, our models considered the effects of trends and seasonality as constant over time, and that may worsen the accuracy of the models, as biosurveillance data often have a substantial temporal structure [39] and fluctuations (e.g., due to a change in government policies, laboratory demands or introduction of a new strain). This issue could be attempted to overcome by implementing an automatic selection of possible distributions from a list [43], or by using more complex approaches such as adaptive regression [37], time-varying autoregressive parameters [44], etc. However, choosing the best regression model in these situations would require more user experience. ARIMA methods are alternatives to address the lack of independence of observations by modelling autocorrelation, but we found very low values for most of the parameters of detection with these models (< 25%). They were also the only ones that improved by adding a guard band, which may suggest overfitting problems. Furthermore, ARIMA and derivatives demand experienced human supervision to identify components, making their use difficult for automatized applications [17, 45]. Thus, our results suggest that other alternatives may be explored before applying ARIMA methods.

Regarding SCC, EARS methods have been widely used in surveillance due to practical advantages such as working with short historical data available [5] and ease of computation, but they are generally outperformed by other SCC or regression-based methods [36], as it was found in our study. The other SCC methods are very sensitive to the temporal structure of a time series (trends, seasons, “day of week”, etc.) so, it is necessary to preprocess the data to obtain a stationary time series [25]. We used different methods of pre-processing and fed CUSUM and Shewhart algorithms with the residuals, finding that LSTM pre-processing generally provided better results. CUSUM methods (*lstm_c*, *far_c* and *breg_c*) generally worked better in terms of Se, but POD was only good in large outbreaks (i.e., > 75% when *k* > 12) when LSTM was used for pre-processing and did not perform well in terms of timeliness and BAR. CUSUM tends to work better detecting sustained changes even if they are smaller [46, 47]. Finally, EWMA is a mixture of SCC and smoothing technique and a superiority in detecting slow shifts in the process mean is expected [6]. However, in our case the performance was in the middle suggesting that other methods could be better alternatives.

When the overall score was assessed, LSTM showed better potential to report anomalies along with Bayesian approaches and *rki* (Figure 4). Machine learning methods such as LSTM may be better to identify hidden patterns in complex data in situations with no clear trends or non-linearity [48], as we have observed in our study. The algorithms based on Bayesian approaches (*bay1–3*) presented high values for POD, Se and POD1w compared to most of the other methods, which is consistent with other studies that compared Bayesian methods with other statistical approaches [35, 42]. However, some of these methods (*lstm_c*, *rki1–2*, and *bay1–2*) presented high BAR values, which hinders their practical use, and even in the anomaly detection methods that performed better, a higher capability of detection would also be desirable.

The novelty of our approach lies in the use of a very specific source of data, sequences, instead of clinical signs or non-specific indicators, which are typically used in SyS [5]. Surveillance schemes based on laboratory results may also be of great benefit in guiding early control through disease-specific interventions [12, 49]. For example, in the context of PRRSV, surveillance based on pre-diagnostic signs may not be very effective, as the typical signs of disease (e.g., abortions, stillbirths, respiratory disease, slow growth rates, lethargy, anorexia, etc.) are nonspecific and are shared with other diseases that may also be endemic in the population. As routine sequencing is increasing in swine production systems in several countries, monitoring, and detection of anomalies can be relatively inexpensive and simple, taking advantage of data already collected. According to our results, the main limitation is a low capability of detection when the size of the outbreak is small. However, at production system level, small outbreaks mean that few farms are involved, so their non-detection, although undesired, may be more tolerated since these emergencies are not posing a great threat to the entire system. Thus, the approach evaluated in this study still could be useful to alert of situations of multiple emergences in farms. This may serve as an early indication to trigger enhanced control measures in the production system, for example, increasing surveillance and better tracking of the movement of infected animals between farms and the introduction of vehicles, fomites, or aerosols, etc. [50–52].

Compared to using other types of data, monitoring sequences to detect new strains generally performed best. Only using time series of PCR positives performed similarly. In the system considered in this study sequencing results can be obtained within a week, but in other systems waiting times can be longer, which could compromise the timeliness of the method. Since the performance measures between PCR positive and new strains were quite similar, the former may be used as a first alarm while waiting for sequencing results or when results are not provided promptly. In contrast, anomaly detection with PCR and laboratory requests resulted less helpful. In these cases, the evaluated methods performed notably worse: lower POD and Se and extremely high BAR in some cases (e.g., *bay2*) (Figure 5). Furthermore, the use of these data sources does not present significant practical advantages since PCR results can be rapidly available. Interestingly, POD and Se were lower in those time series that presented higher SD, so the lack of detectability may be explained because the synthetic cases are masked by the high background noise, and so large injections are required to trigger alarms. Some methods also presented high POD1w, despite low POD and Se, but this is likely an artifact due to the low values of POD favoring extreme values. Approaches based on requests or other unspecific parameters would likely require of more complex approaches or higher outbreaks to be able to detect anomalies among the noise. On the contrary, if they are properly designed, they would allow an earlier detection due to the waiting until diagnosis is not necessary. In this regard, other studies have showed great potential on using anomaly detection in laboratory requests or production data [5, 25, 53, 54]. Using sequence data may have other limitations. For example, sample submission may have little granularity at farm level, as it is generally not economically sustainable on a daily basis, and sequencing can also be less frequent on small farms or at specific production stages, such as finishing farms. This reduces the amount of information from a system that can be captured and tends to delay interventions.

The relatively good performance of sequences compared with other sources of data shows a promising application of surveillance based on sequence data to assess the presence of outbreaks. This could be especially important when syndromic surveillance may be too unspecific. With the increasing use of molecular diagnostics, a valuable source of sequences is available and can be monitored to obtain precise information about the sanitary status of herds and production systems. We have showed the value of this approach compared to other sources of data. Still, there is room to improvement. Further studies incorporating other diseases and outbreak distributions are recommended to gain more insights in alternative scenarios and improve the diagnostic validity of these methods. The algorithms evaluated here can be easily extrapolated to monitor other time series of routinely collected data such as mortality, weight gain, etc., when they can be used as a reliable proxy of the presence of disease. The results of this study will hopefully aid to inform which methods perform well or poorly in this specific setting and contribute to the development of proactive approaches for animal health early detection and rapid control.

### Supplementary Information

**Additional file 1. Overview of the characteristics of the different types of methodologies evaluated in the study.**

**Additional file 2. Number of synthetic cases generated in each synthetic outbreak in 1000 iterations by value of *k.***

**Additional file 3. Duration of synthetic outbreaks in 1000 iterations by value of *k.***

**Additional file 4. Code used for analysis and evaluation presented in the article.**

**Additional file 5. Sample of the data for analysis.**

**Additional file 6. Code to run the methods and algorithms evaluated in the article.**

## Data Availability

The datasets used and/or analyzed during the current study are available from the corresponding author on reasonable request.

## References

[1] World Organization for Animal Health (OIE) (2020) One World, One Health. 2020. https://www.oie.int/app/uploads/2021/03/bull-2009-2-eng.pdf. Accessed 4 May 2022

[2] Rushton J, Gilbert W (2016) The economics of animal health: direct and indirect costs of animal disease outbreaks. In: 84th World Assembly of OIE

[3] European Commision (2007) A new Animal Health strategy for the European Union (2007–2013) where “Prevention is better than cure.” Communication from the commission to the council, the European parliament, the European economic and social committee and the committee of the regions

[4] Dórea FC, Sanchez J, Revie CW (2011). Veterinary syndromic surveillance: current initiatives and potential for development. Prev Vet Med.

[5] Dórea FC, Vial F (2016). Animal health syndromic surveillance: a systematic literature review of the progress in the last 5 years (2011–2016). Vet Med.

[6] Smith GE, Elliot AJ, Lake I, Edeghere O, Morbey R, Catchpole M, Heymann DL, Hawker J, Ibbotson S, McCloskey B, Pebody R (2019). Syndromic surveillance: two decades experience of sustainable systems—its people not just data!. Epidemiol Infect.

[7] Abat C, Chaudet H, Rolain JM, Colson P, Raoult D (2016). Traditional and syndromic surveillance of infectious diseases and pathogens. Int J Infect Dis.

[8] Faverjon C, Berezowski J (2018). Choosing the best algorithm for event detection based on the intended application: a conceptual framework for syndromic surveillance. J Biomed Inform.

[9] Salman MD (2008). Animal disease surveillance and survey systems: methods and applications.

[10] Doherr MG, Audige L (2001). Monitoring and surveillance for rare health-related events: a review from the veterinary perspective. Philos Trans R Soc Lond B Biol Sci.

[11] Dupuy C, Bronner A, Watson E, Reist M, Fouillet A, Calavas D, Hendrikx P, Perrin J (2013). Inventory of veterinary syndromic surveillance initiatives in Europe (Triple-S project): current situation and perspectives. Prev Vet Med.

[12] Kosmider R, Kelly L, Evans S, Gettinby G (2006). A stastistical system for detecting *Salmonella* outbreaks in British livestock. Epidemiol Infect.

[13] Murtaugh MP (2012). Use and interpretation of sequencing in PRRSV control programs Take-home messages.

[14] Holtkamp DJ, Kliebenstein JB, Neumann EJ, Zimmerman J, Rotto HF, Yoder TK, Wang C, Yeske PE, Mowrer CL, Haley CA (2013). Assessment of the economic impact of porcine reproductive and respiratory syndrome virus on United States pork producers. J Swine Health Prod.

[15] Shin GE, Park JY, Lee KK, Ku B, Jeoung H (2022). Recombination between the Fostera MLV-like strain and the strain belonging to lineage 1 of porcine reproductive and respiratory syndrome virus in Korea. Viruses.

[16] Ding Y, Wubshet AK, Ding X, Zhang Z, Li Q, Dai J, Hou Q, Hu Y, Zhang J (2021). Evaluation of four commercial vaccines for the protection of piglets against the highly pathogenic porcine reproductive and respiratory syndrome virus (hp-PRRSV) QH-08 strain. Vaccines.

[17] Unkel S, Farrington CP, Garthwaite PH, Robertson C, Andrews N (2012). Statistical methods for the prospective detection of infectious disease outbreaks: a review. J R Stat Soc.

[18] Shmueli G, Burkom H (2010). Statistical challenges facing early outbreak detection in biosurveillance. Technometrics.

[19] Serfling RE (1963). Methods for current statistical analysis of excess pneumonia-influenza deaths. Public Health Rep.

[20] De Vries A, Reneau JK (2010). Application of statistical process control charts to monitor changes in animal production systems. J Anim Sci.

[21] Farrington CP, Andrews NJ, Beale AD, Catchpole MA (1996). A statistical algorithm for the early detection of outbreaks of infectious disease. J R Stat Soc.

[22] Hulth A, Andrews N, Ethelberg S, Dreesman J, Faensen D, van Pelt W, Schnitzler J (2010). Practical usage of computer-supported outbreak detection in five European countries. Euro Surveill.

[23] Malhotra P, Vig L, Shroff G, Agarwal P (2015) Long short term memory networks for anomaly detection in time series. In: Proceedings, 89. Presses universitaires de Louvain

[24] Disease Bioportal. https://bioportal.ucdavis.edu

[25] Dórea FC, McEwen BJ, McNab WB, Revie C, Sanchez J (2013). Syndromic surveillance using veterinary laboratory data: data pre-processing and algorithm performance evaluation. J R Soc Interface.

[26] Alonso C, Murtaugh MP, Dee SA, Davies PR (2013). Epidemiological study of air filtration systems for preventing PRRSV infection in large sow herds. Prev Vet Med.

[27] Noufaily A, Enki DG, Farrington P, Garthwaite P, Charlett A (2012). An improved algorithm for outbreak detection in multiple surveillance systems. Stat Med.

[28] Pileri E, Mateu E (2016). Review on the transmission porcine reproductive and respiratory syndrome virus between pigs and farms and impact on vaccination. Vet Res.

[29] Xing J, Burkom H, Tokars J (2011). Method selection and adaptation for distributed monitoring of infectious diseases for syndromic surveillance. J Biomed Inform.

[30] Tokars JI, Burkon H, Xing J, English R, Bloom S, Cox K, Pavlin JA (2009). Enhancing time-series detection algorithms for automated biosurveillance. Emerg Infect Dis.

[31] Buckingham-Jeffery E, Morbey R, House T, Elliot A, Harcourt S, Smith GE (2017). Correcting for day of the week and public holiday effects: improving a national daily syndromic surveillance service for detecting public health threats. BMC Public Health.

[32] Rolfhamre P, Ekdahl K (2006). An evaluation and comparison of three commonly used statistical models for automatic detection of outbreaks in epidemiological data of communicable diseases. Epidemiol Infect.

[33] Hutwagner LC, Thompson WW, Seeman GM, Treadwell T (2005). A simulation model for assessing aberration detection methods used in public health surveillance for systems with limited baselines. Stat Med.

[34] Choi BY, Kim H, Go UY, Jeong JH, Lee JW (2010). Comparison of various statistical methods for detecting disease outbreaks. Comput Stat.

[35] Bédubourg G, Le SY (2017). Evaluation and comparison of statistical methods for early temporal detection of outbreaks: a simulation-based study. PLoS ONE.

[36] Jackson ML, Baer A, Painter I, Duchin J (2007). A simulation study comparing aberration detection algorithms for syndromic surveillance. BMC Med Inform Decis Mak.

[37] Wang X, Zeng D, Seale H, Li S, Cheng H, Luan R, He X, Pang X, Dou X, Wang Q (2010). Comparing early outbreak detection algorithms based on their optimized parameter values. J Biomed Inform.

[38] Zhou H, Burkom H, Winston CA, Dey A, Ajani U (2015). Practical comparison of aberration detection algorithms for biosurveillance systems. J Biomed Inform.

[39] Buckeridge DL, Burkom H, Campbell M, Hogan WR, Moore AW (2005). Algorithms for rapid outbreak detection: a research synthesis. J Biomed Inform.

[40] Buckeridge DL (2007). Outbreak detection through automated surveillance: a review of the determinants of detection. J Biomed Inform.

[41] Mandl KD, Reis B, Cassa C (2004). Measuring outbreak-detection performance by using controlled feature set simulations. MMWR Morb Mortal Wkly Rep.

[42] Yuan M, Boston-Fisher N, Luo Y, Verma A, Buckeridge DL (2019). A systematic review of aberration detection algorithms used in public health surveillance. J Biomed Inform.

[43] Dórea FC, Widgren S, Lindberg A (2015). Vetsyn: an R package for veterinary syndromic surveillance. Prev Vet Med.

[44] Held L, Hofmann M, Höhle M, Schmid V (2006). A two-component model for counts of infectious diseases. Biostatistics.

[45] Burkom HS, Murphy SP, Shmueli G (2007). Automated time series forecasting for biosurveillance. Stat Med.

[46] Kass-Hout TA, Xu Z, McMurray P, Park S, Buckeridge DL, Brownstein JS, Finelli L, Groseclose SL (2012). Application of change point analysis to daily influenza-like illness emergency department visits. J Am Med Inform Assoc.

[47] Texier G, Farouh M, Pellegrin L, Jackson ML, Meynard JB, Deparis X, Chaudet H (2016). Outbreak definition by change point analysis: a tool for public health decision?. BMC Med Inform Decis Mak.

[48] Provotar OI, Linder YM, Veres MM (2019) Unsupervised Anomaly Detection in Time Series Using LSTM-Based Autoencoders. In: 2019 IEEE international conference on advanced trends in information theory, ATIT 2019—Proceedings. IEEE, pp 513–517

[49] Hutwagner LC, Maloney EK, Bean NH, Slutsker L, Martin SM (1997). Using laboratory-based surveillance data for prevention: an algorithm for detecting Salmonella outbreaks. Emerg Infect Dis.

[50] Mortensen S, Stryhn H, Sogaard R, Boklund A, Stärk KDC, Christensen J, Willeberg P (2002). Risk factors for infection of sow herds with porcine reproductive and respiratory syndrome (PRRS) virus. Prev Vet Med.

[51] Otake S, Dee SA, Jacobson L, Torremorell M, Pijoan C (2002). Evaluation of aerosol transmission of porcine reproductive and respiratory syndrome virus under controlled field conditions. Vet Rec.

[52] Dee SA, Deen J, Otake S, Pijoan C (2004). An experimental model to evaluate the role of transport vehicles as a source of transmission of porcine reproductive and respiratory syndrome virus to susceptible pigs. Can J Vet Res.

[53] Dórea FC, McEwen BJ, McNab WB, Sanchez J, Revie C (2013). Syndromic surveillance using veterinary laboratory data: algorithm combination and customization of alerts. PLoS ONE.

[54] Merca C, Lindell IC, Ernholm L, Selling L, Nunes T, Sjölund M, Dórea F (2022). Veterinary syndromic surveillance using swine production data for farm health management and early disease detection. Prev Vet Med.

[55] Pedregosa F, Varoquaux G, Gramfort A, Michel V, Thirion B, Grisel O, Blondel M, Prettenhofer P, Weiss R, Dubourg V, Vanderplas J, Passos A, Cournapeau F, Brucher M, Perrot M, Duchesnay E (2011). Scikit-learn: machine learning in python. J Mach Learn Res.

[56] Abadi M, Agarwal A, Barham P, Brevdo E, Chen Z, Citro C, Corrado GS, Davis A, Dean J, Devin M, Ghemawat S, Goodfellow I, Harp A, Irving G, Isard M, Jia Y, Jozefowicz R, Kaiser L, Kudlur M, Levenberg J, Mane D, Monga R, Moore S, Murray D, Olah C, Schuster M, Shiens J, Steiner B, Sutskever I, Talwar K, et al. (2016) TensorFlow: large-scale machine learning on heterogeneous systems arXiv:1603.04467v2

[57] Brooks ME, Kristensen K, van Benthem KJ, Magnusson A, Berg CW, Nielsen A, Skaug HJ, Mächler M, Bolker BM (2017). glmmTMB balances speed and flexibility among packages for zero-inflated generalized linear mixed modeling. R J.

[58] Salmon M, Schumacher D, Höhle M (2016). Monitoring count time series in R: aberration detection in public health surveillance. J Stat Softw.

[59] Riebler A (2004) Empirischer Vergleich von statistischen Methoden zur Ausbruchserkennung bei Surveillance Daten. University of Munich

[60] Hyndman RJ, Khandakar Y (2008). Automatic time series forecasting: the forecast Package for R. J Stat Softw.

[61] Liboschik T, Fokianos K, Fried R (2017). tscount: an R package for analysis of count time series following generalized linear models. J Stat Softw.

[62] Scrucca L (2004). qcc: An R package for quality control charting and statistical process control. R News.

[63] Iturria A, Carrasco J, Charramendieta S, Conde A, Herrera F (2020). otsad: A package for online time-series anomaly detectors. Neurocomputing.

[64] Raza H, Prasad G, Li Y (2015). EWMA model based shift-detection methods for detecting covariate shifts in non-stationary environments. Pattern Recognit.

