# Supplementary material for: Evaluation of the application of sequence data to the identification of outbreaks of disease using anomaly detection methods
Source: Vet Res. 2023 Sep 8;54:75. doi: 10.1186/s13567-023-01197-3 (PMC10492347; doi:10.1186/s13567-023-01197-3)
Supplement: Supplementary file 1 — Additional file 1. Overview of the characteristics of the different types of methodologies evaluated in the study. [file 13567_2023_1197_MOESM1_ESM.docx]

**Overview of the characteristics of the different types of methodologies evaluated in the study.**

**Regression methodologies**. Generalized linear models can be adapted to fit different distributions: linear, log-linear, Poisson, quasi-Poisson, logistic, Negative binomial, or Binomial regression. In time series, regression techniques are based on Serfling’s approach [19]. This is a regression model that controls trends by including a lineal term; and seasonal variations by including sines and cosines. Additional explanatory variables can be added in the model to control for different effects in the time series (e.g., holidays). In the anomaly detection context, regression approaches predict the expected outcome based on time series samples, and contrast that with the observed data. Observed points highly deviated from the predictions are considered anomalies. For forecasting, a reference model is created and used to predict a window of values in the future (expected values), which are compared with the actual values observed. When expected and observed values are different, an anomaly is possible, but there are elements such as the error of the prediction or the background noise of the time series that can interfere in this interpretation. Therefore, in order to determine whether this difference is due to random normal variation or a true anomaly, it is required that the difference overpasses a defined threshold. Threshold values that trigger an alarm are usually set as a multiple of the standard error of the prediction. In general, a value between 2- and 3.5-times the standard error is usually chosen. However, Farrington et al. [21] also proposed exceedance scores based on the upper limit of the prediction interval, which has been subsequently revised [26]. The correlation between observations in time series may be an issue. For more details see [8, 42].

**Bayesian approaches**. Bayesian approaches extend the regression techniques to a Bayesian context. Thus, the posterior distribution of the parameter is estimated considering uncertainty from the observed data and prior knowledge. Currently, there are two approaches: quantifying the probability of being in an epidemic phase (e.g., Hidden Markov Models) or of observing events (e.g., regression).

A simpler approach uses the quantile values of the predictive posterior distribution to define a threshold. In the algorithms used in our paper, these reference values are taken from time windows previous to the period that it is being assessed for the presence of anomalies. The algorithm “*bayes*” in the package R *surveillance* [50] uses a simple conjugate prior-posterior approach and computes the parameters of a negative binomial distribution based on past values. This method does not offer the inclusion of a linear trend, down-weighting of past outbreaks or power transformation of the data.

**Machine learning approaches.** Like the other approaches, Machine learning anomaly detection aims to create accurate baselines of the normal behavior of the time series to identify anomalous values that deviate from these predictions. The core of machine learning is data and loss functions. Models are trying to learn features from the data with the help of loss functions that generalize our models to previously unseen data. Machine learning is based on statistical learning theory and aims to find a function that maps the outcome by using an algorithm that learn what is the best way to approach the problem. This is provided by something called a loss function and then empirically decide whether the loss function is better or not, i.e., empirical risk. A learning algorithm chooses the function that minimizes this empirical risk

In our paper, we explore the use of the Long Short-Term Memory (LSTM) networks. They are a type of recurrent neural network capable of learning order dependence in sequence prediction problems. Because of this characteristic, LSTM perform well in frameworks with recurrent and dependent data, e.g., speech recognition, natural language processing tasks, and time series forecasting. LSTMs have the ability to learn the relationship between past data and current values. The capacity of LSTM to model this dependency has led to a large academic interest in using these units in machine learning for time series forecasting. Similarly, thresholds are set by larger deviations of observed respecting to predicted data.

Machine learning pros are that they do not assume a distribution for the data and try a bunch of different models instead to compare with a predefined regression model. However, the algorithm has a tendency to cheat in order to minimize its loss function by overfitting to data. This is why after learning a function based on the training set data, that function is validated on a test set of data, data that did not appear in the training set.

**Time series methodologies**. They are extensions from regression methodologies used for modeling autocorrelated observations. Like regression techniques, thresholds for alerts are usually set as deviations of the observed values from the predicted values by between 2 and 4 times the standard error. ARMA models have two components (p,q): one for the autoregression (AR) and another for the moving average (MA). This approach has been extended into ARIMA (Autoregressive Integrated Moving Average) models, which present a third component (integrated) (p, d, q) used to remove the trend prior to fitting the MA and AR coefficients. It exists another variation called SARIMA, which is similar to ARIMA but focuses on seasonal variations instead of trends. When a trend or seasonal effects are present in the time series, we have to use ARIMA or SARIMA. In addition, ARIMAX models include additional terms for explanatory variables.

Estimating autocorrelation can only be done on a stationary time series. Therefore, pre-processing of the time series may be necessary to run these methods, i.e., the time series needs to be converted to a format that accomplish the assumptions required by the analytical method. The statistical testing is usually based on the normal distribution, but some approaches exist to adapt it to other types of observations, for example, counts [66].

In ARIMA models, we can predict the predictable components (i.e., trend, seasonality and other factors included in ARIMAX), but not the unpredictable terms (observed variation in the time series, which has not been explained by a factor). Holt-Winters approach can handle this by smoothing the noise by splitting the time series into several parts (a level term, a trend term, and a seasonality term, defined by the smoothing constants α, β, and γ) and combines the results back together afterwards. This method is a triple exponential smoothing that involves exponentially decreasing the weights of observations over time, such that oldest observations have the smallest weight. The forecast is continuously revised according to more recent observations, as past observations are weighted in an exponentially decreasing order, i.e., the most recent observations are given higher weights than old values. A problem of approaches based on smoothing may be a tendency to overfit.

**Statistic Control Charts**. Also called statistical process control, they are methods developed to control and monitor the quality of a process or product, e.g., in manufactured lines, but they may be applied to any process that produce an output that can be measured (e.g., positives to a disease), and they have been used for syndromic surveillance. In this context, the process is the normal presence of disease and outbreaks are understood as an anomaly regarding this normal trend. For calculation. these methods use the mean over a selected baseline period as a measure of expectation and evaluate the differences between data observed in a time window and a threshold which is set at some multiple of the standard deviation of the sample’s mean. There are a number of algorithms that follow this approach. Essentially, all of them derived from the first developed by Shewhart in 1931. Two of the basic assumptions of the early control charts are that the data should be stationary and normally distributed. This implies that time series that do not follow these assumptions need to be transformed to the appropriate distribution (pre-processing). Some of the most widely used methods are: EARS, CUSUM, Shewhart or EWMA. They made different adjustments for specific purposes aiming to get a better performance. For example, changing the length of the window to set the reference sample’s mean, including moving sample averages and sample standard deviation to standardize each observation, include smoothing parameters, etc. and can work better in some contexts or others. For further information [8].

*References may be found in the reference list of the main article*
