# Supplementary figures and images for: Evaluation of the application of sequence data to the identification of outbreaks of disease using anomaly detection methods
Source: Vet Res. 2023 Sep 8;54:75. doi: 10.1186/s13567-023-01197-3 (PMC10492347; doi:10.1186/s13567-023-01197-3)

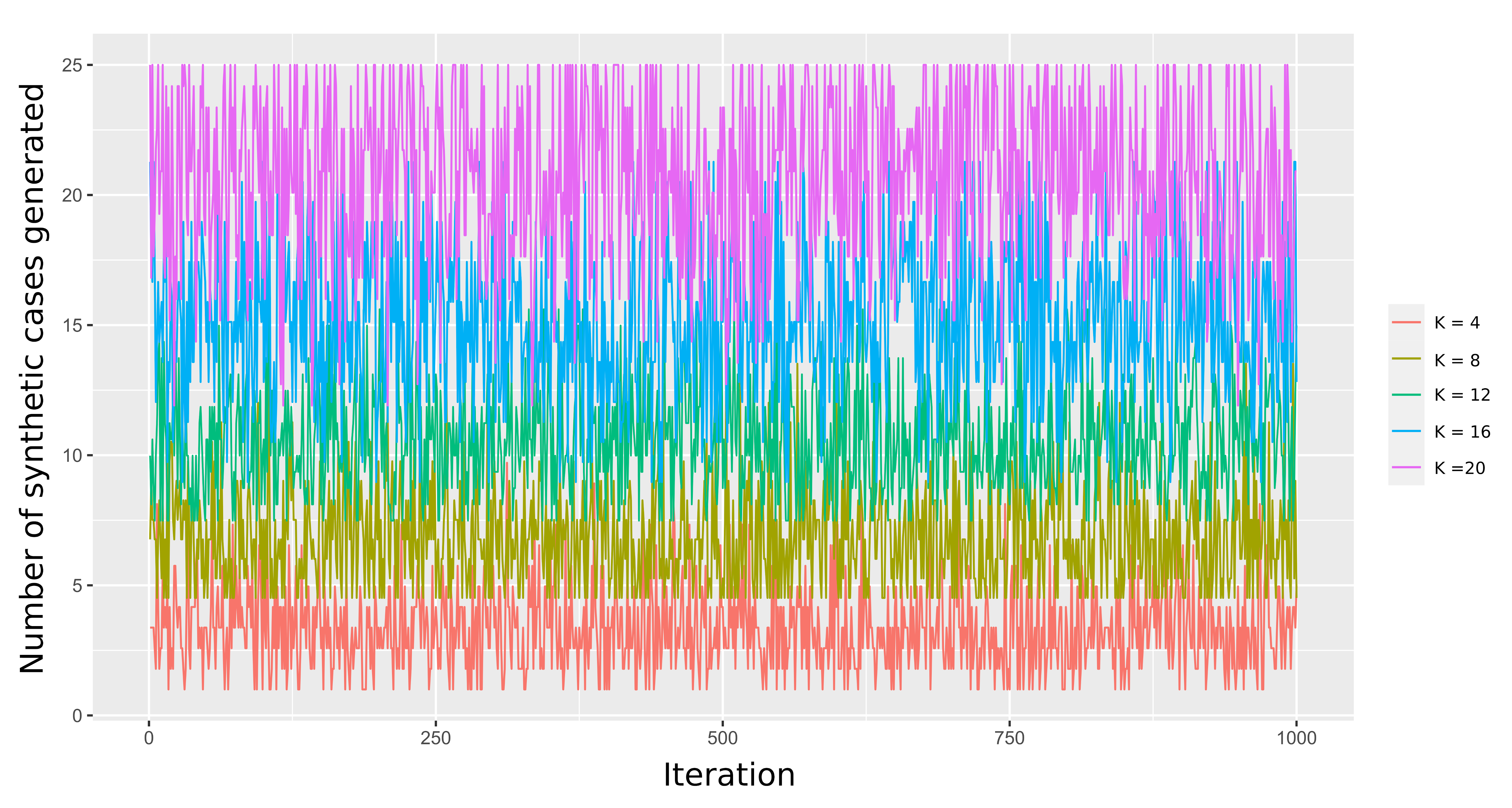

Supplement: Supplementary file 2 — Additional file 2. Number of synthetic cases generated in each synthetic outbreak in 1000 iterations by value of k. [file 13567_2023_1197_MOESM2_ESM.tif]

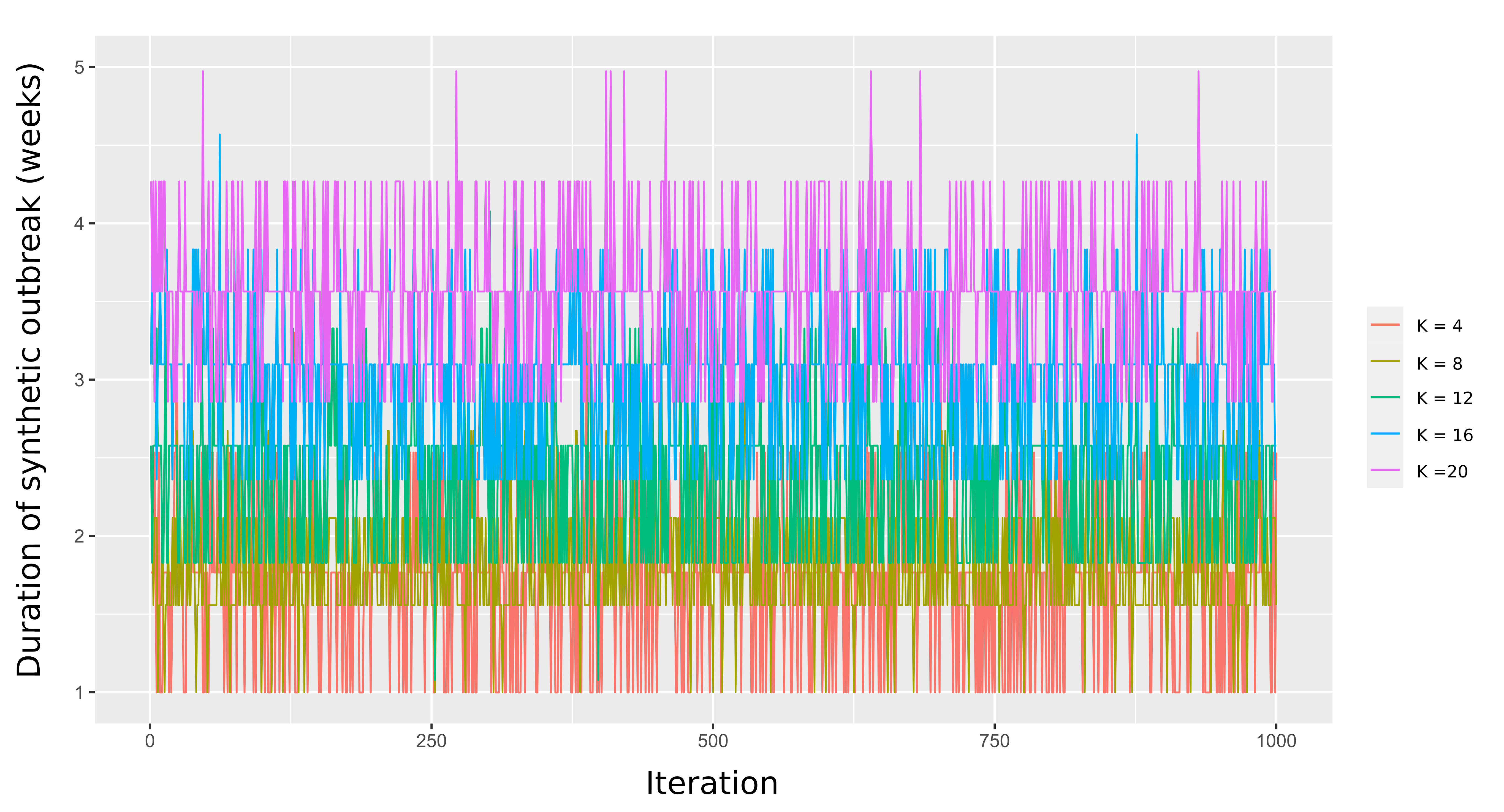

Supplement: Supplementary file 3 — Additional file 3. Duration of synthetic outbreaks in 1000 iterations by value of k. [file 13567_2023_1197_MOESM3_ESM.tif]
